# The Role of Autophagy in Genome Stability through Suppression of Abnormal Mitosis under Starvation

**DOI:** 10.1371/journal.pgen.1003245

**Published:** 2013-01-31

**Authors:** Aiko Matsui, Yoshiaki Kamada, Akira Matsuura

**Affiliations:** 1 Department of Nanobiology, Graduate School of Advanced Integration Science, Chiba University, Inage-ku, Chiba, Japan; 2 National Institute for Basic Biology, Okazaki, Aichi, Japan; The University of North Carolina at Chapel Hill, United States of America

## Abstract

The coordination of subcellular processes during adaptation to environmental change is a key feature of biological systems. Starvation of essential nutrients slows cell cycling and ultimately causes G1 arrest, and nitrogen starvation delays G2/M progression. Here, we show that budding yeast cells can be efficiently returned to the G1 phase under starvation conditions in an autophagy-dependent manner. Starvation attenuates TORC1 activity, causing a G2/M delay in a Swe1-dependent checkpoint mechanism, and starvation-induced autophagy assists in the recovery from a G2/M delay by supplying amino acids required for cell growth. Persistent delay of the cell cycle by a deficiency in autophagy causes aberrant nuclear division without sufficient cell growth, leading to an increased frequency in aneuploidy after refeeding the nitrogen source. Our data establish the role of autophagy in genome stability through modulation of cell division under conditions that repress cell growth.

## Author Summary

A nutrient stress such as nitrogen depletion induces pleiotropic responses in eukaryotic cells. For example, nutrient starvation slows cell cycling and ultimately causes G1 arrest. In addition, it is known that nitrogen starvation delays G2/M progression. However, the mechanism as to how G2/M-delayed cells progress through the cell cycle to return to the G1 phase remains unclear. Cells subjected to a nutrient stress induce autophagy, a bulk degradation system within lysosomes/vacuoles, to reconstitute cellular components. In this study, we show that an autophagy-dependent supply of amino acid pools is critical for completion of cell cycle under starvation conditions in the budding yeast *Saccharomyces cerevisiae*. Autophagy deficiency causes a defect in cell growth and leads to abnormal mitosis associated with a higher incidence of aneuploidy. Thus, our data establish the role of autophagy in genome stability through modulation of cell division under conditions that repress cell growth, which provides a possible mechanism of tumor suppression by autophagy shown in mammalian cells.

## Introduction

Autophagy is a protein degradation pathway that is conserved from yeast to mammals. Previous studies have reported the functions and molecular mechanisms of autophagy, including the identification of autophagy-related genes [1], [2], the characterization of the molecular mechanisms of each autophagy stage [3], [4], and the detection of selective autophagy [5], [6]. Autophagy is induced in response to nutrient starvation. In addition, homeostatic autophagy occurs at a low level in mammalian cells under nutrient-rich conditions [7], [8], and is regulated in a cell cycle-dependent manner [9], [10]. Furthermore, autophagy is induced during developmental stages [11].

In budding yeast, cell division and cell growth are precisely regulated by an intrinsic mechanism, and are tightly modulated by nutrient conditions. Target of rapamycin (TOR) is a phosphatidylinositol kinase-related Ser/Thr kinase and a critical regulator of cell growth, which senses the nutrient conditions [12]. TOR forms two distinct complexes, TOR complex 1 (TORC1) and TOR complex 2 (TORC2) [13]. Yeast TORC1 consists of either of the two yeast TOR homologs, Tor1 or Tor2, together with co-factors Kog1, Lst8, and Tco89, which are sensitive to inhibition by rapamycin. Conversely, yeast TORC2, which is produced from Tor2, Avo1–3, and Lst8, is not sensitive to rapamycin. While TORC2 regulates spatial aspects of cell growth, such as the organization of the actin cytoskeleton, TORC1 regulates temporal aspects of cell growth, including protein synthesis, gene transcription, ribosome biogenesis, amino acid uptake, and induction of autophagy [13]–[16]. Under nutrient-rich conditions, TORC1 is active and inhibits the induction of autophagy through inhibitory phosphorylation of Atg13 [17]. In contrast, TORC1 is inactive during starvation, thereby inducing autophagy [18]. Inhibition of TORC1 by rapamycin or by nutrient starvation leads to cell cycle arrest in the G1 phase [19], demonstrating that TORC1 plays a crucial role linking cell growth and cell cycle progression.

In addition to its role in the regulation of cell cycle progression in the G1 phase, we recently found that TORC1 is involved in the G2/M transition in budding yeast [20]. Reduced TORC1 activity, caused by nutrient starvation or by temperature-sensitive mutation in *KOG1*, which is an essential component of TORC1, leads to cell cycle arrest at G2/M. Two recent reports have shown that nutrient starvation also blocks the onset of mitosis in mammalian cells and the fission yeast *Schizosaccharomyces pombe* [21], [22]. The regulation is achieved by TORC1-mediated activation of Cdc2/cyclin B in mammalian cells [21], whereas it is suggested to be mediated by TORC2 as well as Sty1 MAPK signaling in the fission yeast [22], [23]. Although the underlying mechanisms may differ among species, the transient suppression of mitotic entry in response to nutrient starvation is conserved throughout evolution. However, it is well-known that cells arrest in the G1 phase and enter G0 during starvation [24]. Thus, the mechanism as to how G2/M-delayed cells progress through the cell cycle to return to the G1 phase remains unclear.

In this study, we elucidated the molecular mechanism underlying cell cycle progression under nutrient-limited conditions. We show that the cell cycle delay at G2/M is rescued in an autophagy-dependent manner. Regulation of the cell cycle by autophagy during starvation is believed to be involved in genome integrity by coupling cell growth with cell division.

## Results

### Reduced TORC1 activity by nitrogen starvation is partially recovered in an autophagy-dependent manner

**Figure 1:**
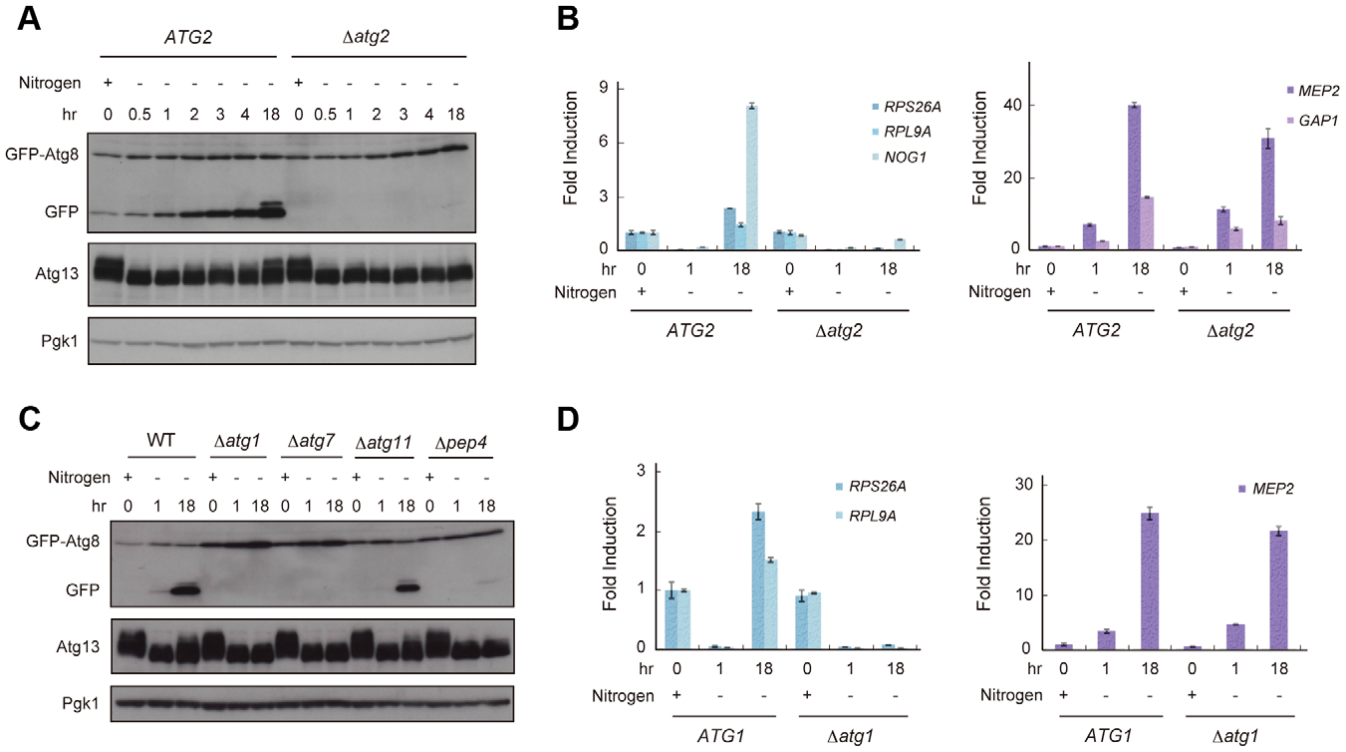
Reduced TORC1 activity by nitrogen starvation is partially recovered in an autophagy-dependent manner. (A) *ATG2* (AMY182-10C) and Δ*atg2* (AMY182-10A) cells grown in SCD-Ura-Trp medium at 30°C were transferred to SD-N medium for the indicated times. Cell lysates were prepared using the alkaline-trichloroacetic acid method and analyzed by immunoblot with anti-GFP, anti-Atg13 and anti-Pgk1 antibodies. Progression of autophagy was monitored by accumulation of the free GFP moiety from the GFP-Atg8 fusion protein [65]. Pgk1 was used as a loading control. Uncropped images of blots are shown in Figure S5. Samples taken at 4-h intervals for 20 h demonstrated that the re-phosphorylation of Atg13 increased monotonically (data not shown). (B) *ATG2* (AMY182-10C) and Δ*atg2* (AMY182-10A) cells grown in SCD-Ura-Trp medium at 30°C were transferred to SD-N medium for the indicated times. Total RNA was extracted and analyzed for the expression of *RPS26A*, *RPL9A* and *NOG1* (left panel), and *MEP2* and *GAP1* (right panel) by RT-qPCR. Each sample was calibrated by *TUB1* (Figure S1). (C) WT (AMY182-10C), Δ*atg1* (AMY236), Δ*atg7* (AMY237), Δ*atg11* (AMY238), and Δ*pep4* (AMY239) cells grown in SCD-Ura-Trp medium at 30°C were transferred to SD-N medium for the indicated times. Cell lysates were prepared and analyzed by immunoblot as described in (A). Uncropped images of blots are shown in Figure S5. (D) *ATG1* (SAY122) and Δ*atg1* (AMY240) cells grown in SCD-Ura-Trp medium at 30°C were transferred as described in (C). Total RNA was extracted and analyzed for the expression of *RPS26A* and *RPL9A* (left panel) and *MEP2* (right panel) by RT-qPCR. Each sample was calibrated by *TUB1*.

TORC1 is thought to be a nutrient sensor that controls the cell cycle and autophagy directly [12], [18]. We first examined fluctuation in TORC1 activity under starvation conditions by an immunoblot of Atg13, a TORC1 substrate, and RT-qPCR of genes whose expression is a product of TORC1 function. TORC1 directly phosphorylates Atg13 [17], thus, inactivation of TORC1 causes dephosphorylation of Atg13. In addition, TORC1 induces the transcription of *RPS26A*, *RPL9A*, and *NOG1* but suppresses that of *MEP2* and *GAP1* [16], [25], [26]. When exponentially growing wild-type (WT) cells were released into nitrogen-depleted medium, TORC1 activity was immediately decreased; Atg13 became dephosphorylated (Figure 1A), resulting in a concomitant decrease in the expression of *RPS26A*, *RPL9A*, and *NOG1* and an increase in the expression of *MEP2* and *GAP1* (Figure 1B). We found that phosphorylation of Atg13 gradually recovered after 2–18 h in nitrogen-depleted medium (Figure 1A), suggesting that TORC1 activity was restored in cells. The expressions of *RPS26A*, *RPL9A*, and *NOG1* were increased, but those of *MEP2* and *GAP1* remained at high levels 18 h after release into nitrogen-depleted medium (Figure 1B). The lack of a correlation between the recovery of Atg13 phosphorylation and the levels of *MEP2* and *GAP1* suggests that TORC1 activity did not completely recover during this process.

Next, we investigated whether autophagy is involved in the recovery of TORC1, and assessed the fluctuation in TORC1 activity in autophagy-deficient Δ*atg2* cells. When Δ*atg2* cells were placed in nitrogen-depleted medium, TORC1 activity decreased, similar to WT cells up to 4 h (Figure 1A and 1B). Interestingly, TORC1 activity was not restored in Δ*atg2* cells 18 h after being released into the starvation medium (Figure 1A and 1B, left panel).

Re-phosphorylation of Atg13 and increased expression of *RPS26A* and *RPL9A* under starvation conditions required other autophagy-related genes, such as *ATG1* and *ATG7*, which are essential for autophagosome formation, and *PEP4* encoding vacuolar proteinase A, which is responsible for autophagic degradation of proteins accompanied by recycling of amino acids (Figure 1C and 1D, left panel). In contrast, recovery of TORC1 activity was not affected by deletion of *ATG11*, which is essential in selective autophagy and dispensable for starvation-induced autophagy (Figure 1C). Therefore, these results suggest that the partial recovery of TORC1 activity is induced in a non-selective and starvation-induced autophagy-dependent manner.

### Autophagy is required for cell cycle progression during starvation

The cell cycle is arrested in the G1 phase under starvation conditions [19], and we previously reported that TORC1 inactivation caused by rapamycin treatment and nitrogen starvation induces a G2/M delay [20]. Therefore, we postulated that an unknown mechanism may exist that regulates cell cycle re-progression from G2/M under nutrient starvation. We carefully examined the cell cycle profiles of nitrogen-starved cells, particularly the relationship between cell cycle progression and TORC1 activity.

**Figure 2:**
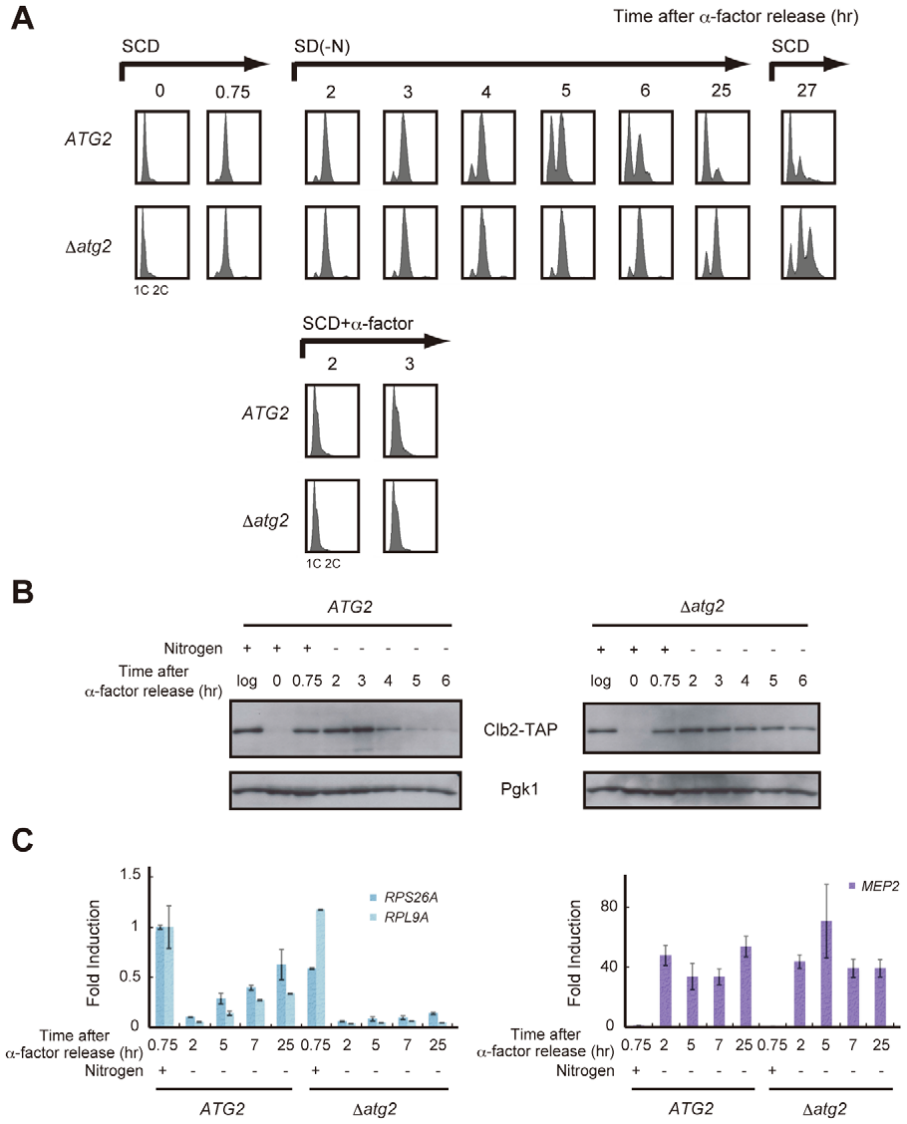
Autophagy is required for cell cycle progression during starvation. (A) *ATG2* (SAY122) and Δ*atg2* (AMY250) cells were arrested at G1 by α-factor and released into SCD medium. Synchronous cultures were collected after 0.75 h and re-released into SD-N medium or SCD medium. To monitor cell cycle progression to G1 under the nutrient-rich condition, SCD medium was supplemented with 6.7 ng/mL α-factor for re-arrest at G1. After 25 h, the cell culture was re-released into SCD medium. DNA content at each time point was measured by FACS analysis. (B) *ATG2* (AMY251) and Δ*atg2* (AMY253) cells were arrested at G1 by α-factor and released into SCD medium. Synchronous cultures were collected after 0.75 h and re-released into SD-N medium. Cell lysates were prepared as described in Figure 1A, and analyzed by immunoblot with anti-PAP and anti-Pgk1 antibodies. Pgk1 was used as a loading control. Uncropped images of blots are shown in Figure S5. (C) *ATG2* (SAY122) and Δ*atg2* (AMY250) cells were grown as described in (A). Total RNA was extracted and analyzed for the expression of *RPS26A* and *RPL9A* (left panel) and *MEP2* (right panel) by RT-qPCR. Each sample was calibrated by *TUB1*.

First, WT and Δ*atg2* cells were synchronized in the G1 phase by treatment with α-factor, and released into SCD medium. When the majority of cells progressed to the S phase, they were released into nitrogen-depleted medium. During this time course, cells were collected at intervals and the DNA content of cells at each time point was examined by FACS analysis. As previously reported, WT and Δ*atg2* cells remained arrested at 2C DNA content for 2–4 h after α-factor release, demonstrating that cell cycle progression was delayed at G2/M (Figure 2A). In WT cells, the delay in cell cycle progression was overcome after 5 h, and most cells reached the G1 phase after 25 h (Figure 2A). In contrast, a signification portion of Δ*atg2* cells, as well as Δ*atg1* cells, remained arrested at 2C DNA content after 25 h (Figure 2A and Figure S2). The difference in the cell cycle profiles between WT and *atg2* mutant cells was confirmed by Clb2 levels; G2/M cyclin Clb2 decreased 4–5 h after α-factor release in WT cells, indicating that the cells entered mitosis, whereas Clb2 was consistently present in Δ*atg2* cells (Figure 2B). These results show that autophagy contributes to re-progression at G2/M during starvation.

Next, we investigated whether re-activation of TORC1 is correlated with re-progression at G2/M. We analyzed time-dependent changes in gene expression downstream of TORC1. In WT cells, transcription of *RPS26A* and *RPL9A* decreased in response to starvation (2 h), but was gradually restored after 5–25 h (Figure 2C). Mitotic entry correlated with the re-activation of TORC1 (Figure 2A and 2C). Conversely, transcription of *RPS26A* and *RPL9A* remained low in Δ*atg2* cells, and the cells remained arrested at G2/M (Figure 2A and 2C). These results show again that TORC1 is partially restored in an autophagy-dependent manner in nitrogen-starved cells, and suggest that a correlation between the partial recovery of TORC1 activity and cell cycle re-progression at G2/M.

### A specific amino acid supply is sufficient for cell cycle re-progression after a G2/M delay

To further address the mechanism of autophagy-dependent cell cycle re-progression during starvation, we next focused on the role of amino acid pools. Autophagy contributes to the maintenance of amino acid pools in yeast; during the first two hours of nitrogen starvation, the intracellular amino acid level decreases rapidly, and is then partially recovered in an autophagy-dependent manner [27], [28]. Since the amino acids produced by enhanced autophagy are utilized for new protein synthesis [27], [28], we investigated whether specific amino acids produced by autophagy contribute to cell cycle re-progression after a G2/M delay.

**Figure 3:**
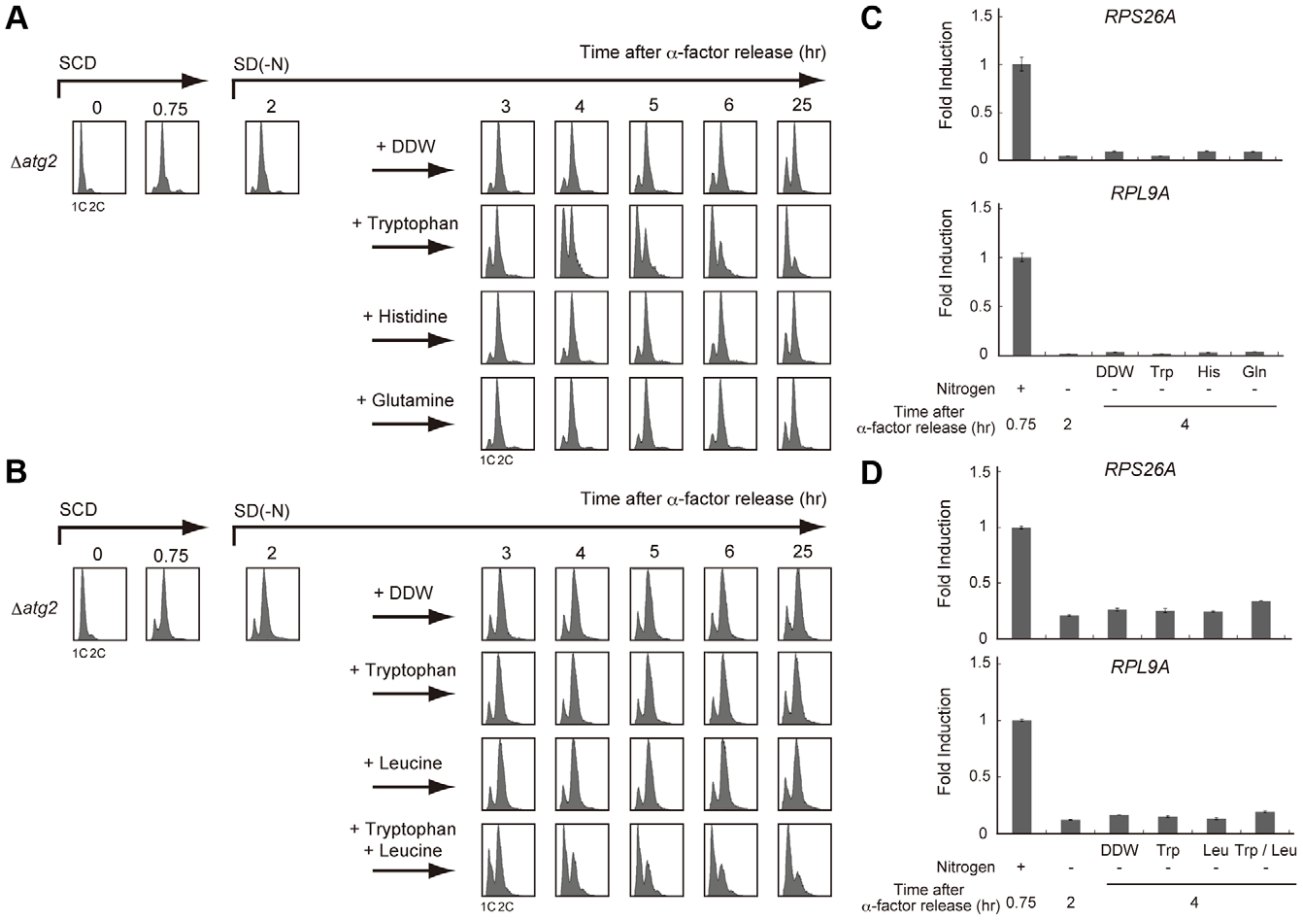
Supply of specific amino acids is sufficient for cell cycle re-progression after G2/M delay. (A) Δ*atg2* (AMY250) cells were arrested at G1 by α-factor and released into SCD medium. Synchronous cultures were collected after 0.75 h and re-released into SD-N medium. After 2 h from α-factor release, each culture was supplemented with tryptophan, histidine, or glutamine at a final concentration of 25 µg/mL, 10 µg/mL and 25 µg/mL, respectively. Double distilled water (DDW) was used as a control. DNA content at each time point was measured by FACS analysis. (B) Δ*atg2* (AMY296) cells were arrested at G1 by α-factor and released into SCD medium. Synchronous cultures were collected after 0.75 h and re-released into SD-N medium. After 2 h from α-factor release, each culture was supplemented with tryptophan, leucine, or both tryptophan and leucine. DDW was used as a control. DNA content at each time point was measured by FACS analysis. Tryptophan and leucine were used at a final concentration of 25 µg/mL and 10 µg/mL, respectively. (C) Δ*atg2* (AMY250) cells were grown as described in (A). Total RNA was extracted and analyzed for the expression of *RPS26A* (upper panel) and *RPL9A* (lower panel) by RT-qPCR. Each sample was calibrated by *TUB1*. (D) Δ*atg2* (AMY296) cells were grown as described in (B). Total RNA was extracted and analyzed for the expression of *RPS26A* (upper panel) and *RPL9A* (lower panel) by RT-qPCR. Each sample was calibrated by *TUB1*.

The Δ*atg2* strain AMY250 possesses *his3*, *trp1*, and *ura3* mutations causing auxotrophies for histidine, tryptophan, and uracil, respectively. We postulated that these auxotrophic mutations would affect the intracellular pools of the corresponding amino acids especially upon starvation. As hypothesized, the G2/M-delayed Δ*atg2* mutant returned to G1 phase similarly to wild-type (WT) cells when tryptophan was added to the nitrogen-starved medium (Figure 3A). In contrast, the addition of histidine did not affect the suppression of the prolonged G2/M delay in the Δ*atg2* mutant, nor did the addition of glutamine, the level of which is believed to be involved in TORC1 activity in yeast [29] (Figure 3A).

Recent studies have shown that TORC1 activity is regulated by the availability of some amino acid species including leucine; this is dependent on the editing function of aminoacyl-tRNA transferase [30], [31]. Using another Δ*atg2* mutant (AMY296), which is congenic to AMY250 with adenine and leucine auxotrophies, we further examined the effect of amino acid supplementation to a starvation medium on autophagy-deficient cells. As shown in Figure 3B, the addition of tryptophan alone was insufficient for the cell cycle progression of AMY296. However, the simultaneous addition of tryptophan and leucine efficiently rendered the cells to a G1 arrest. These results show that cell cycle perturbations caused by a deficiency in autophagy may be suppressed by the addition of amino acids specific to the strains, suggesting that autophagy contributes to cell cycle progression by allocating some, but not all, amino acids that are present in a limited intracellular amount.

Next, we examined whether the addition of specific amino acids would upregulate the starvation-repressed TORC1 activity. Interestingly, while the addition of amino acids mimicked cell cycle progression in the autophagy-proficient cells, it was not associated with an increase in transcription of TORC1 downstream *RPS26A* and *RPL9A* (Figure 3C and 3D). This result raises the possibility that TORC1 recovery is not essential for cell cycle re-progression after a G2/M delay, although TORC1 re-activation correlates with re-progression.

### The recovery of TORC1 activity may be dispensable for cell cycle progression under starvation conditions

**Figure 4:**
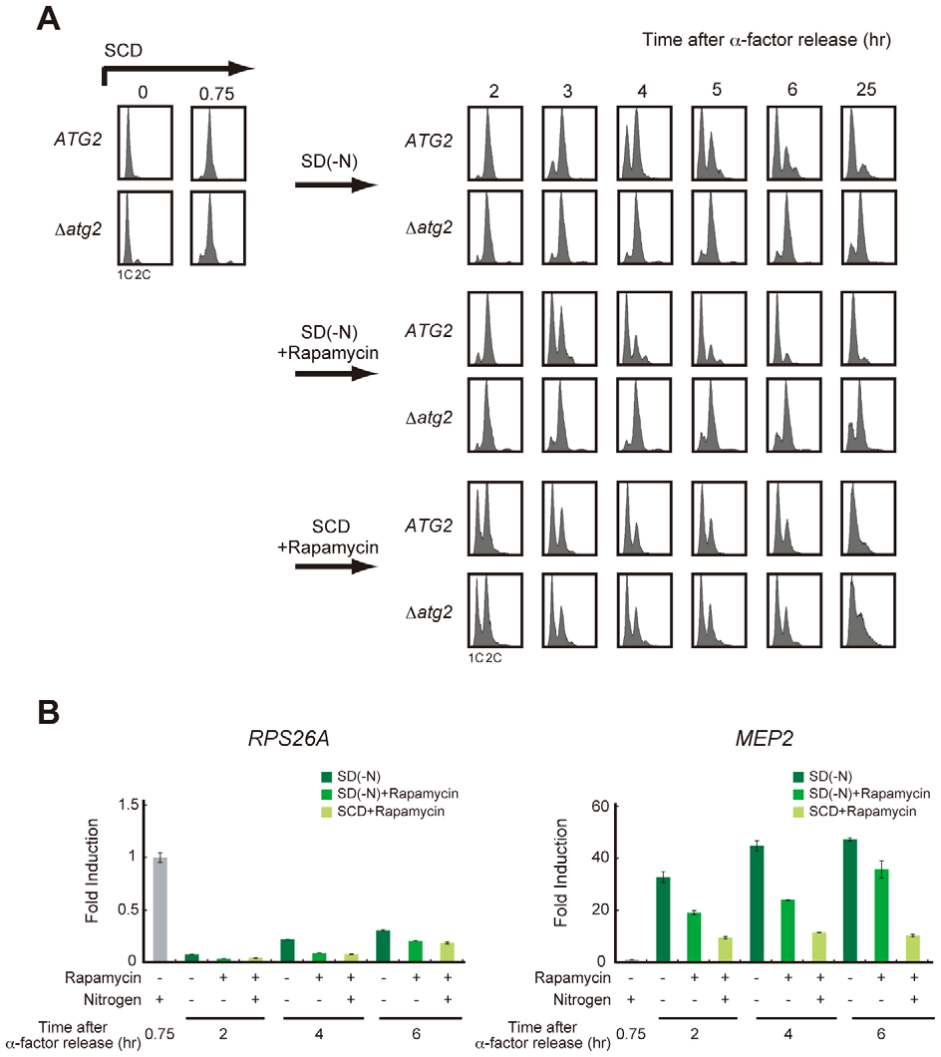
Recovery of TORC1 activity may be dispensable for cell cycle progression under starvation conditions. (A) *ATG2* (SAY122) and Δ*atg2* (AMY250) cells were arrested at G1 by α-factor and released into SCD medium. Synchronous cultures were collected after 0.75 h and re-released into SD-N medium, SD-N medium containing rapamycin, or SCD medium containing rapamycin. DNA content was measured by FACS analysis. Rapamycin was used at a final concentration of 200 ng/mL. (B) *ATG2* (SAY122) cells were grown as described in (A). Total RNA was extracted and analyzed for the expression of *RPS26A* (left panel) and *MEP2* (right panel) by RT-qPCR. Each sample was calibrated by *TUB1*.

To examine the causal relationship between the recovery of TORC1 activity and cell cycle re-progression, we examined cell cycle progression during starvation when TORC1 activity was inhibited by the addition of rapamycin. As reported previously [20], rapamycin treatment transiently delayed cell cycle progression at G2/M in nutrient-rich SCD medium, and most cells returned to G1 irrespective of their autophagic competency (Figure 4A). In contrast, rapamycin-treated WT cells showed a G2/M delay and proceeded to G1 when rapamycin was added to the nitrogen-starved medium (Figure 4A). TORC1 activity, monitored by *RPS26A* expression, was persistently lower in rapamycin-treated cells than that in those without rapamycin treatment during starvation (Figure 4B), supporting the hypothesis that TORC1 recovery is dispensable for cell cycle re-progression after a G2/M delay. Cell cycle progression to G1 phase in rapamycin-treated starved cells was significantly faster than in those that without rapamycin treatment (Figure 4A). However, the effect of rapamycin was not observed in Δ*atg2* cells, in which the cell cycle remained arrested in the G2/M transition in nitrogen-starved medium, even with the addition of rapamycin (Figure 4A). Again, these results show that autophagy is critical for cell cycle progression from G2/M to G1 under starvation conditions, and suggest the involvement of autophagy-dependent supply of amino acid pools in the accelerated cell cycle progression by rapamycin treatment.

We further examined the role of TORC1 activity in cell cycle re-progression using a temperature-sensitive *KOG1*mutant (*kog1*-*105*) [20], encoding an essential component of TORC1. Since the *kog1-105* mutant showed a G2/M arrest phenotype at the restrictive temperature, the experiment was performed at 25°C, a permissive temperature at which the mutant is able to proliferate. When cells were cultured under nitrogen-starved conditions, the G2/M delay was prolonged in *kog1*-*105* cells compared to WT cells (Figure S3A). During the course of the experiment, TORC1 activity monitored by *RPS26A* expression was slightly lower in the *kog1-105* mutant than in WT cells, both of which were exposed to nutrient-rich condition and nitrogen-starved conditions (Figure S3B). Moreover, the delay in cell cycle re-progression in the *kog1-105* cells was relieved by the addition of rapamycin (Figure S3C). This result shows that further inhibition of TORC1 activity in *kog1-105* cells by rapamycin is sufficient for cell cycle re-progression at G2/M, suggesting that the delay in cell cycle re-progression of the *kog1-105* mutant is not likely due to the reduction in global TORC1 activity. Rather, *kog1-105* may cause a defect in specific pathways downstream of TORC1, and the residual activity of TORC1 in the *kog1-105* mutant, which can be inhibited by rapamycin, may prevent cell cycle recovery after a G2/M delay.

### Autophagy is important for normal cell division and cell growth during nutrient starvation

**Figure 5:**
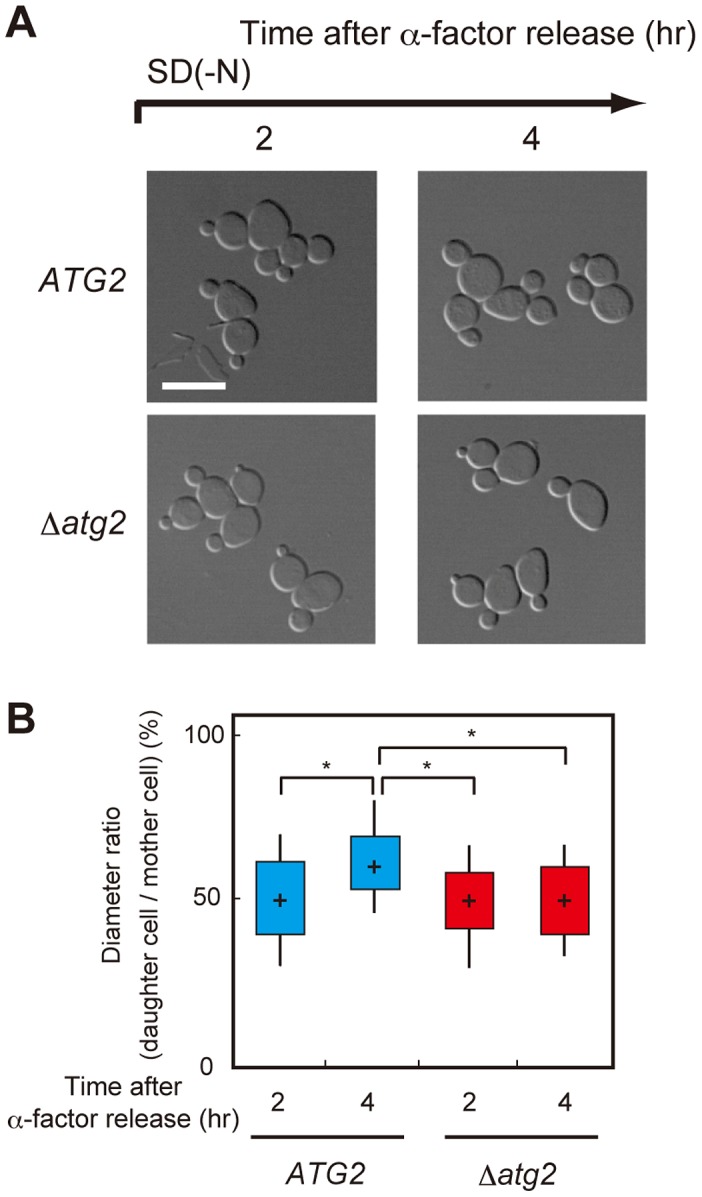
Autophagy is important for cell growth under nutrient starvation conditions. (A) *ATG2* (SAY122), Δ*atg2* (AMY255) cells were arrested at G1 by α-factor and released into SCD medium. Synchronous cultures were collected after 0.75 h and re-released into SD-N medium. The fixed cells were observed by differential interference contrast microscopy. Bar, 10 µm. (B) *ATG2* (SAY122) and Δ*atg2* (AMY255) cells were grown as described in (A). The ratio of the diameter of a bud or daughter cell to that of the mother was calculated 2 h and 4 h after α-factor release. The box plots represent the distribution of the ratios. The plot shows the medians (central cross figure) with the 25th and 75th percentiles (box). The lower line protruding from the box ends at the 10th percentiles, whereas the upper line ends at the 90th percentile. Single asterisks designate significant differences (*P*<0.01); unstarred comparisons are not significantly different (*P*>0.05).

We investigated how amino acids produced by autophagy contribute to re-progression of the cell cycle from a G2/M delay under starvation conditions. We first observed the morphology of WT and Δ*atg2* cells cultured under starvation conditions. As shown in Figure 5, the daughter cell of Δ*atg2* cells was smaller than that of WT cells 4 h after α-factor release, suggesting that amino acids produced by autophagy are important for sufficient cell growth during nutrient starvation.

**Figure 6:**
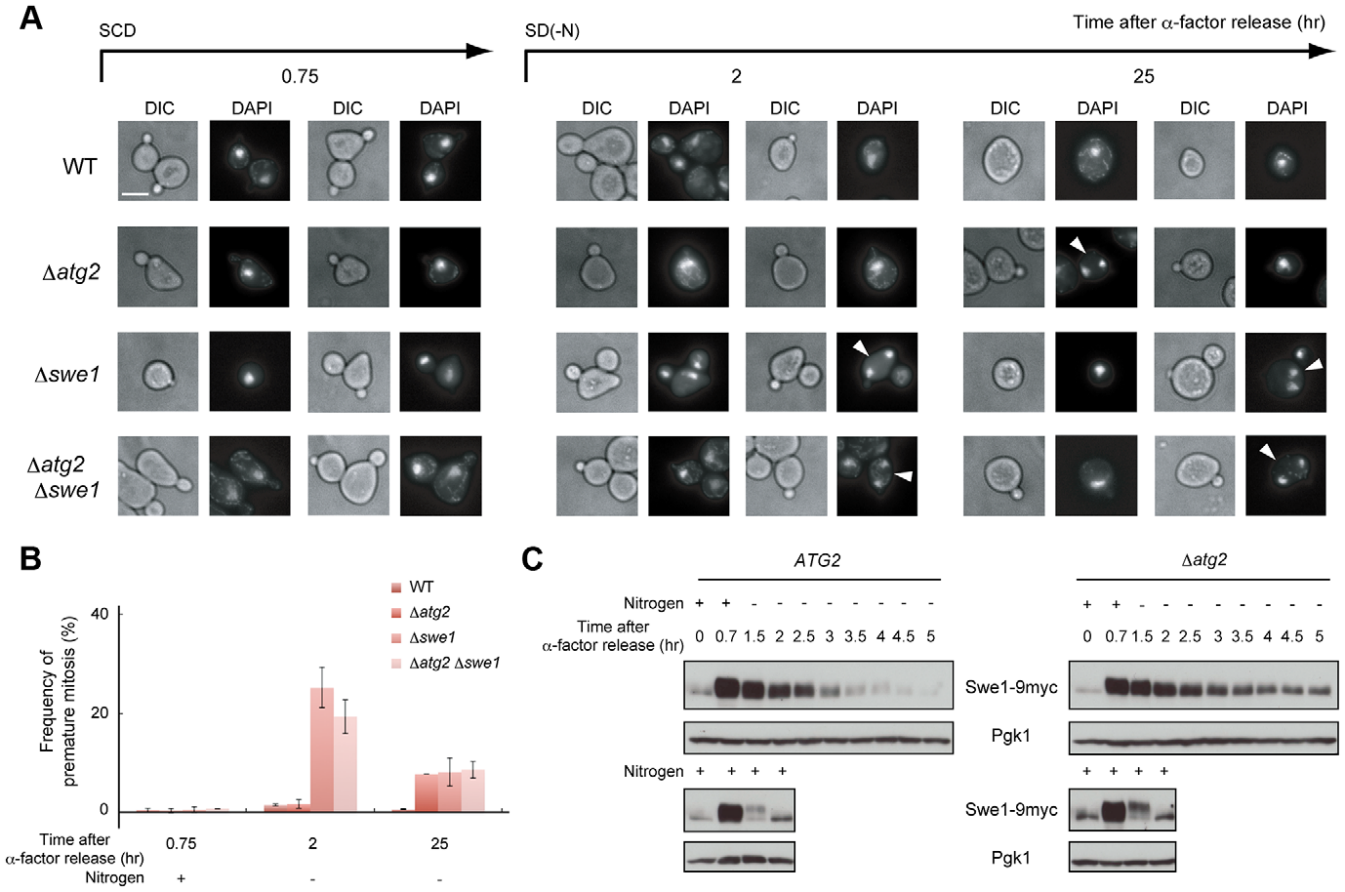
Autophagy is important for normal cell division under nutrient starvation conditions. (A) WT (SAY122), Δ*atg2* (AMY255), Δ*swe1* (AMY260), and Δ*atg2* Δ*swe1* (AMY261) cells were arrested at G1 by α-factor and released into SCD medium. Synchronous cultures were collected after 0.75 h and re-released into SD-N medium. The fixed cells were stained with DAPI and observed by differential interference contrast microscopy (DIC) or by fluorescence microscopy (DAPI). The arrowheads indicate cells undergoing premature mitosis (type 4 in Figure 7A). Bar, 5 µm. (B) WT (SAY122), Δ*atg2* (AMY255), Δ*swe1* (AMY260), and Δ*atg2* Δ*swe1* (AMY261) cells were grown as described in (A). The frequency of premature mitosis (type 4 in Figure 7A) was calculated. (C) *ATG2* (YYK536) [66] and Δ*atg2* (AMY330) cells were arrested at G1 by α-factor and released into SCD medium. Synchronous cultures were collected after 0.7 h and re-released into SD-N medium or SCD medium. To monitor cell cycle progression to G1 under the nutrient-rich condition, SCD medium was supplemented with 6.7 ng/mL α-factor for re-arrest at G1. Cell lysates were prepared as described in Figure 1A, and analyzed by immunoblot with anti-myc and anti-Pgk1 antibodies. Pgk1 was used as a loading control. Uncropped images of blots are shown in Figure S5.

**Figure 7:**
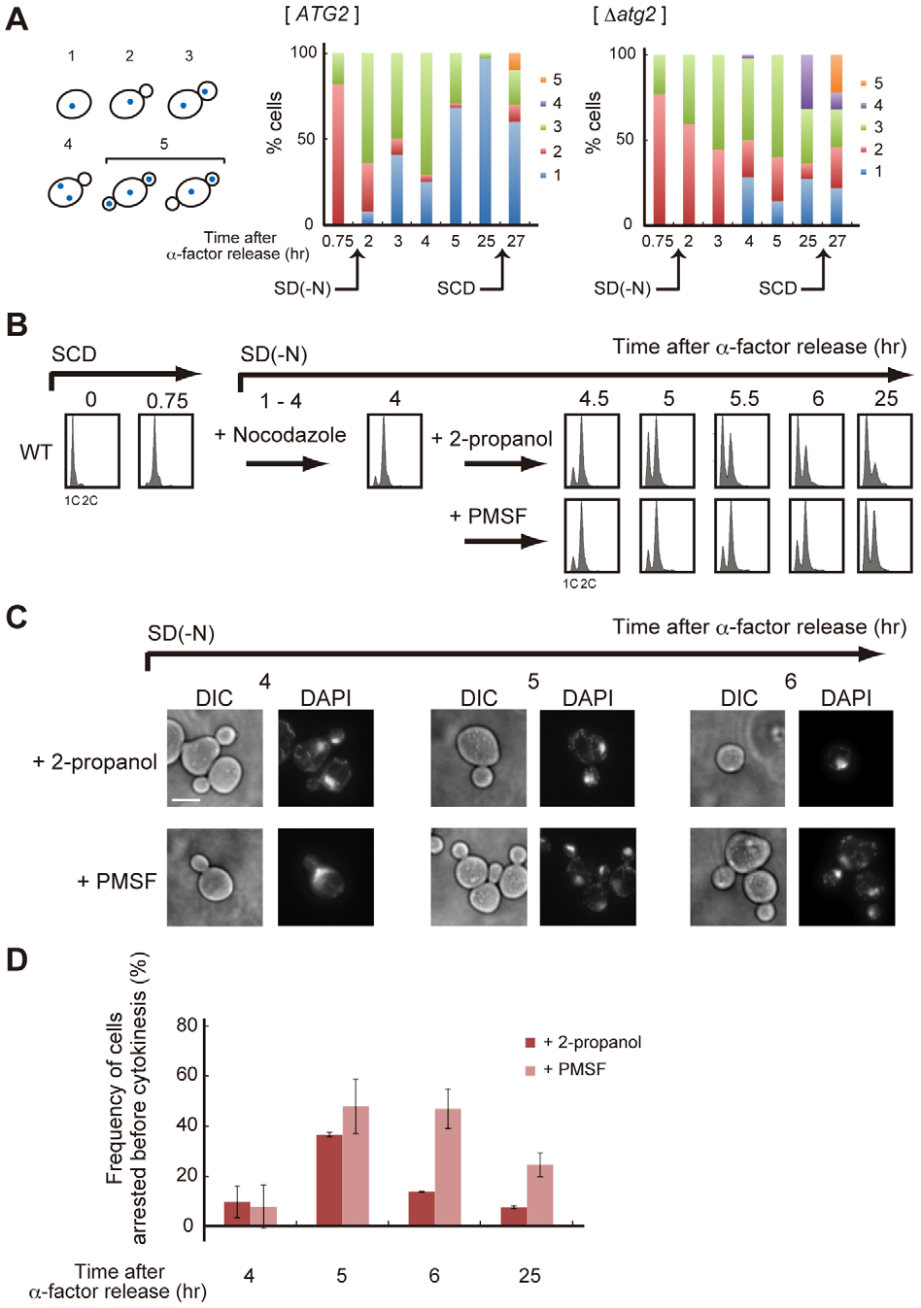
Autophagy is required for completion of cytokinesis. (A) *ATG2* (SAY122) and Δ*atg2* (AMY250) cells were arrested at G1 with α-factor and released into SCD medium. Synchronous cultures were collected after 0.75 h and re-released into SD-N medium. After 25 h, cell cultures were re-released into SCD medium. Cells were observed by differential interference contrast microscopy, and Hoechst 33342-stained nuclei and the tubulin-GFP of each cell were observed by fluorescence microscopy. The rates of cells with the following morphologies were calculated: 1) unbudded cells with a nucleus, 2) small-budded cells with a nucleus, 3) large-budded cells with one nucleus each in the mother and the daughter, 4) small-budded cells with two nuclei in the mother, and 5) cells harboring two small buds, with one nucleus each in the mother cells and at least one daughter cell. (B) WT (SAY122) cells were arrested at G1 by α-factor and released into SCD medium. Synchronous cultures were collected after 0.75 h and re-released into SD-N medium containing 5 µg/mL nocodazole. After 3 h, synchronous cultures arrested at metaphase by nocodazole were collected and re-released into SD-N medium containing 1 mM PMSF. 2-propanol was used as a control. DNA content at each time point was measured by FACS analysis. (C) WT (SAY122) cells were grown as described in (B). The fixed cells were stained with DAPI and observed by differential interference contrast microscopy (DIC) or by fluorescence microscopy (DAPI). Bar, 5 µm. (D) WT (SAY122) cells were grown as described in (B). The frequency of cells arrested before cytokinesis (type 3 in A) was calculated.

The status of the bud (daughter cell) and timing of nuclear division are strictly regulated by the Swe1-dependent checkpoint mechanism in budding yeast [32]–[35]. Therefore, we further analyzed cell division using Δ*swe1* and Δ*atg2* Δ*swe1* cells by DAPI staining to examine the potential relationship between the checkpoint mechanism and autophagy. As shown in Figure 6A and 6B, loss of the Swe1 function in both WT and Δ*atg2* cells caused an increase in premature mitosis at early time points (2–3 h after α-factor release; type 4 in Figure 7A). Interestingly, some of the Δ*swe1* cells arrested at 2C DNA content underwent normal cell division and returned to the G1 phase after 25 h (Figure S4), suggesting that premature mitosis caused by Swe1 dysfunction was rescued. This result is likely due to the spindle orientation checkpoint [36], which can correct an abnormal nuclear position at later time points.

Since the Swe1-dependent checkpoint delays entry into mitosis by regulating Swe1 degradation [37], we examined the amount of Swe1 in response to nitrogen starvation. During normal cell cycling under nutrient-rich conditions, Swe1 was accumulated 40 min after α-factor release, and degraded after 1.5 h (Figure 6C). Under starvation conditions, Swe1 was more stable, and degraded only after 3 h (Figure 6C). These results support the hypothesis that the starvation-induced delay of mitosis is mediated by Swe1. This notion is consistent with our previous result that TORC1 regulates G2/M progression through budding yeast polo-like kinase Cdc5, a negative regulator of Swe1 in the initiation of mitosis [20].

In Δ*atg2* cells, degradation of Swe1 occurred normally in nutrient-rich medium, and was delayed under starvation conditions (Figure 6C). Indeed, in Δ*atg2* cells, cells that underwent premature mitosis were scarcely observed 2 h after α-factor release (Figure 6A and 6B). Degradation of Swe1 was delayed in Δ*atg2* cells, and a portion of Swe1 still remained 5 h after α-factor release (Figure 6C), showing that autophagy is important for Swe1 degradation in nutrient-starved cells. However, we noted that Swe1 decreased gradually in Δ*atg2* cells under starvation conditions. Consistently, after 2–3 h, approximately half of the Δ*atg2* cells were arrested before mitosis, but at later time points, the majority of the cells appeared to enter mitosis, as indicated by a reduction in the population of cells with one nucleus (type 2 in Figure 7A). Thus, autophagy is important for efficient recovery from the Swe1-dependent checkpoint under starvation conditions, although autophagy-deficient cells might, at least partly, execute nuclear division after a prolonged cell cycle delay. Nonetheless, cells cannot complete cell division without autophagy under starvation conditions because, unlike Δ*swe1* cells, the majority of Δ*atg2* Δ*swe1* cells remained arrested at 2C DNA content even 25 h after α-factor release (Figure S4). Indeed, cells that passed nuclear division but not cytokinesis (type 3 in Figure 7A) also accumulated transiently in WT cells 2–4 h after α-factor release, and such cells were consistently observed even at later time points in Δ*atg2* cells (Figure 7A).

To examine whether autophagy is involved in cell cycle progression after nuclear division, we used an anti-microtubule drug nocodazole to synchronize cell cycle at metaphase. WT cells were first synchronized in the G1 phase by treatment with α-factor, released into SCD medium, and then transferred into nitrogen-depleted medium containing nocodazole. After 3 h when the majority of cells was arrested at metaphase, cells were collected and re-released into SD-N medium containing 1 mM PMSF that specifically inhibited autophagic degradation under nutrient-starved conditions [38]. As shown in Figure 7B–7D, nocodazole-arrested cells showed a delay in completion of cytokinesis when autophagy was inhibited by the addition of PMSF. Therefore, autophagy contributes not only to nuclear division, but also to cytokinesis, or cell separation, under nutrient-starved conditions.

### Autophagy deficiency results in an increased frequency of aneuploidy after the addition of a nitrogen source

**Figure 8:**
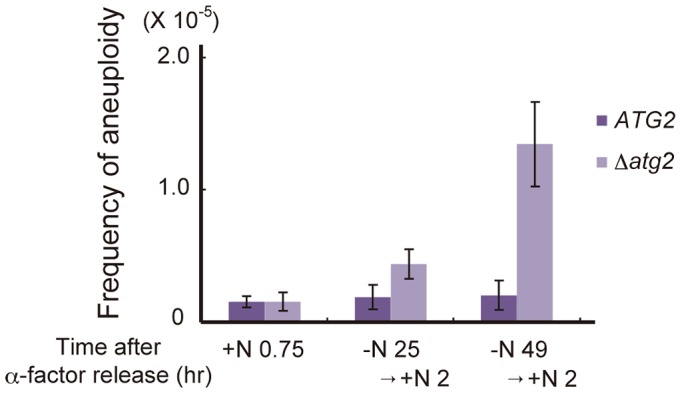
Autophagy deficiency results in an increased frequency of aneuploidy after the addition of a nitrogen source. *ATG2* (DBY4962) and Δ*atg2* (AMY262) cells arrested at G1 by α-factor were released into SC-Ura medium. Synchronous cultures were collected after 0.75 h and re-released into SD-N medium. After 25 h and 49 h, cell cultures were re-released into SCD medium. Appropriately diluted 0.75, 25+2 and 49+2 h aliquots were plated onto YEPD plates and selective medium lacking both leucine and uracil. Colonies were scored after 4 days at 30°C.

Finally, we investigated the physiological significance of autophagy-dependent cell cycle re-progression from G2/M to G1 during starvation conditions. Δ*atg2* cells cultured in nitrogen-starved conditions for 24 h showed an increased frequency of aberrant mitosis, in which two nuclei were present in a mother cell (type 4 in Figure 7A; Figure 6A and 6B). This was likely due to the leaky recovery from the Swe1-dependent mitotic delay. Moreover, when cells were replenished with a nitrogen source and cultured for 2 h, cells with unusually high DNA content (3C DNA content) appeared in Δ*atg2* and Δ*atg1* mutants but not in WT cells (Figure 2A and Figure S2). In addition, analysis by a genetic system using intrachromosomal recombination [39] revealed that Δ*atg2* cells cultured in starvation medium displayed an increased frequency of aneuploidy (2.3-fold and 6.6-fold higher than that of WT cells after starvation for 24 and 48 h, respectively; Figure 8). These results indicate that cell cycle arrest in the G1 phase under starvation conditions is critical for the normal progression of mitosis after restoration of nutrient conditions, and highlight the importance of autophagy-dependent cell cycle re-progression in genome stability.

## Discussion

Although nutrient starvation reduces TORC1 activity and subsequently induces cell cycle arrest in the G1 phase [19], cell cycle progression at the G2/M boundary was blocked under starvation conditions [20]–[23]. Autophagy has been implicated in cellular physiologies under starvation conditions, and the data presented herein uncover another aspect of autophagic functions, namely, its contribution to cell cycle regulation and genome integrity in nutrient-starved yeast cells through the regulation of mitosis progression.

Nuclear division and cytokinesis are two critical events during the cell cycle, both of which require protein synthesis [40]. Here we showed that, in addition to nuclear division, cytokinesis is another step of the cell cycle with limitations, whose entry is blocked or slowed by nutrient starvation, and that starvation-induced autophagy is required to overcome the cell cycle delay at both steps (Figure 6 and Figure 7). Previous reports have revealed that amino acids produced by autophagy are used for protein synthesis [27], [28]. In the present study, we showed that the defects in cell cycle re-progression caused by a lack of autophagy was suppressed by the addition of particular amino acids (Figure 3), and that autophagy promoted the growth of daughter cells in starvation medium (Figure 5). These results clearly show that the amino acid supply through autophagy contributes to sufficient cell growth during nutrient starvation. We found that this phenotype was associated with auxotrophies to specific amino acids, including leucine and tryptophan, but not histidine or nucleobases, including uracil and adenine (Figure 3). The composition of the amino acid pool in budding yeast cells is influenced by nutrient conditions; cells using NH_4_^+^ as the sole nitrogen source accumulate glutamic acid and arginine, but contain amino acids such as tyrosine, leucine, tryptophan, and phenylalanine at low levels [41]. Thus, we assume that, at least under our experimental conditions, leucine and tryptophan are limiting amino acids whose pools cannot support protein synthesis without autophagy upon nitrogen starvation. It will be interesting to examine the potential difference in amino acid requirements for the suppression of autophagy deficiency by varying the composition of amino acid pools using alternate nitrogen sources. In cultured human cells, the cellular pools of glutamine and glutamic acid are maintained at high levels, whereas those of tryptophan, cysteine, and arginine are maintained at low levels [42]. It is important to note that the latter three are essential amino acids that cannot be synthesized *de novo* in humans; thus, autophagy may be involved in maintaining the pools of these amino acids in human cells.

A recent paper reported that TORC1 activity was partially reactivated in an autophagy-dependent manner in ongoing starvation conditions, which played a role in the attenuation of autophagy [43]. Consistent with their findings, we demonstrated that starvation-induced autophagy leads to partial re-activation of TORC1 activity and that the timing of the re-activation correlates with that of cell cycle re-progression at G2/M (Figure 2). Although a reduction in TORC1 activity appears to contribute to the transient cell cycle delay at G2/M, we showed that the cell cycle delay was relieved under TORC1-repressed conditions (Figure 4). These results argue against the positive role of TORC1 re-activation during a rescue from the delay. Although TORC1 activity is involved in translation [19], previous studies have shown that the synthesis of specific proteins continues under TORC1-inhibited conditions [44]. Thus, even in case that TORC1 activity is not re-activated, protein synthesis supported by the autophagy-mediated amino acid pools may be sufficient for starvation-adapted cells to complete a final round of the cell cycle.

Although the inhibition of TORC1 by rapamycin did not abolish cell cycle re-progression from a G2/M delay, the temperature-sensitive *kog1-105* mutation did affect this process (Figure 4 and Figure S3). There are three possible models to explain the cell cycle-specific phenotype of *kog1-105* cells. In the first model, *kog1-105* cells may be defective in only a part of multiple TORC1 functions, and the function affected by *kog1-105* is necessary for cell cycle progression after a G2/M delay. In the second model, TORC1 may be significantly affected in *kog1-105* and the level of TORC1 activity in the mutant is below that caused by nutrient starvation or rapamycin treatment in WT cells. The last model, based on the possibility that *kog1-105* is a gain-of-function mutation, suggests that Kog1-105 protein fulfills a function in addition to the TORC1 function, which is not performed by the normal Kog1 protein. Our results showing that rapamycin suppresses the *kog1-105* defect contradict the latter two models; therefore, it is likely that the *kog1-105* mutation blocks only a portion of the pathways downstream of TORC1. This scenario is consistent with our previous findings that *kog1-105* is not defective in the progression of G1 and does not induce autophagy under nutrient-rich conditions at a restrictive temperature [20]. We also found that the *kog1-105* mutation affected the interaction between Kog1 and TORC1 [20]. Raptor, the mammalian ortholog of Kog1, contributes to the inhibition of mTOR activity upon nutrient depletion through stabilization of the mTOR-Raptor complex [45]. Although it is unknown whether Kog1 inhibits TORC1 under starvation conditions, a decreased interaction with TORC1 could contribute to the phenotypes observed in *kog1-105*, which are distinct from those caused by rapamycin treatment.

There are several examples of rapamycin-insensitive, TORC1-dependent processes in mammalian cells [46]. Although the presence of such processes is elusive in yeast, it is possible that rapamycin-insensitive TORC1 activity is involved in the recovery from a G2/M delay. However, under these circumstances, it is difficult to explain why the addition of rapamycin to nitrogen-depleted medium leads to an early recovery from a cell cycle delay (Figure 4A). It is interesting to note that the addition of rapamycin to fission yeast cells leads to early mitotic onset in nutrient-rich medium, resembling the cell cycle behavior caused by a reduction in the quality of the nutrient source [47]. In budding yeast, cellular responses to rapamycin are not identical to those induced by nitrogen starvation [48], [49]; rapamycin treatment rapidly activates the quality-sensitive nitrogen discrimination pathway, which is distinct from the nitrogen starvation pathway, to facilitate use of poor nitrogen sources. Such a difference may induce rapid adaptation to nitrogen starvation in rapamycin-treated cells. Indeed, the addition of rapamycin facilitates adaptation to an environment containing a low-quality nitrogen source in budding yeast [50]. We tested the involvement of the nitrogen discrimination pathway in the rapamycin-induced early cell cycle recovery by deleting *GLN3*, which encodes the key transcriptional regulator in the nitrogen discrimination pathway, and found that rapamycin treatment accelerated recovery from the G2/M delay even in Δ*gln3* mutants (data not shown). Therefore, we can at least conclude that rapamycin-induced transcription through Gln3 is not essential for this phenotype. It is known that TORC1 regulates a variety of cellular events including transcription, translation, and post-translation [51]. Note that autophagy is essential for rapamycin-mediated early cell cycle recovery (Figure 4A) and that protein synthesis supported by the amino acid pool appears to be involved in this mechanism. Further studies are required to specify the pathway responsible for the early recovery of cell cycle by rapamycin. In addition, it would be interesting to determine physiological conditions that induce the early cell cycle recovery phenotype in a similar manner to that caused by rapamycin treatment; such approaches will help clarify the significance of cell cycle regulation in response to acute inhibition of TORC1 activity.

We have shown that the G2/M specific function of TORC1 is mediated by the polo-like kinase Cdc5 [20], an upstream regulator of Swe1 [52]. Swe1 is involved in a checkpoint mechanism to ensure accuracy of cell division by monitoring daughter cells [32]–[35]. When this checkpoint mechanism is inhibited by the *swe1* mutation, or overexpression of its negative regulators, *HSL1* and *HSL7*, nuclear division occurs prematurely, even if the growth of the bud is suppressed by a mutation in *CDC24* [53]. We observed a similar premature mitosis of Δ*swe1* cells under nitrogen starvation (Figure 5A and 5C), indicating that the Swe1-dependent checkpoint, probably activated by insufficient bud growth, contributes to the G2/M delay phenotype induced by starvation. We found that a deficiency in *SWE1* also increased the fraction of cells that cannot return to the G1 phase normally (Figure S4), indicating that both the Swe1-dependent cell cycle delay and the autophagy-dependent recovery are critical for the integrity of mitosis. Thus, timely regulation of cell cycle progression is of significance under starvation conditions.

Although autophagy is required for the growth of daughter cells during starvation conditions (Figure 5), in our experimental conditions, more than 80% of the autophagy-deficient cells ultimately proceeded to nuclear division after delay at the G2/M boundary. This may be because cells can produce a limited pool of amino acids that are independent of autophagy that support maturation of the bud to overcome the Swe1-dependent checkpoint. Otherwise, long-term starvation might cause an imbalance in the amount of proteins regulating M phase entry, thereby initiating nuclear division, since the Swe1-dependent checkpoint does not appear to monitor bud size per se, but detects an accumulation of mitotic cyclins in the bud [54]. In either case, cell division is not completed before cytokinesis without autophagy. Cytokinesis may serve as an additional control gate for the fulfillment of daughter cell maturation under nutrient-limited conditions.

Cell cycle-dependent regulation of constitutive autophagy has been shown in mammalian cells [7]–[10]. However, this study established the direct involvement of autophagy in cell cycle regulation under starvation conditions; autophagy ensures the accomplishment of the final round of cell cycle progression to nutritionally starved cells. Without autophagy, prolonged treatment with nitrogen starvation caused perturbation of the cell cycle, including premature mitosis, and caused an increased frequency of aneuploidy in budding yeast (Figure 6A, 6B and Figure 8). Thus, in addition to the developmental significance of returning to the G1 phase under starvation conditions (i.e., meiotic division is only initiated from the G1 phase in diploid yeast cells), our results indicate that returning to the G1 phase is critical for maintaining genome integrity after restoration of the nutrient condition from starvation. Notably, a previous study reported that compromised autophagy promotes genomic instability, such as increased DNA damage, gene amplification, and aneuploidy in mammalian cells [55], consistent with the tumor-suppressive activity of autophagy that was previously reported [56]–[59].

Our results demonstrate that autophagy allows a final round of cell cycle progression in budding yeast cells by supplying amino acids during nutrient starvation. This type of regulation can be considered part of the feedback system that maintains the minimum cell volume after division. Asymmetric division, the manner of cell division in the budding yeast, would require sufficient *de novo* protein synthesis for producing a daughter cell different from the mother, and autophagy may support this under nutrient-starved conditions. It will be interesting to determine whether autophagy-dependent regulation is implicated in genetically determined asymmetric division in mammalian development, such as differentiation and maintenance of stem cells. Further studies will shed light on the physiological roles of autophagy, including cell cycle regulation and development.

## Materials and Methods

### Strains, plasmids, media, and genetics

The yeast strains used in this study are listed in Table S1. Unless otherwise noted, all strains were derived from W303-1A and W303-1B. DBY4962 was a gift from Dr. Botstein (Princeton University, USA) [39]. Standard genetic techniques including growth media, cell growth conditions, and transformations were performed as described previously [60], [61].

The plasmid, pRS316-*GFP*-*ATG8*, was a gift from Dr. Suzuki (University of Tokyo, Japan). It harbors the *ATG8* sequence with an N-terminal GFP tag in pRS316 [62]. The *GFP*-*ATG8* fragment was integrated into pRS314 to construct pRS314-*GFP*-*ATG8*. YEp352-*ATG13*, pRS313-*KOG1*, and pRS313-*kog1*-*105* were described previously [20].

### Immunoblotting

Samples corresponding to 0.2 OD_600_ (anti-GFP, anti-Pgk1, PAP, and anti-myc antibodies) or 0.1 OD_600_ (anti-Atg13 antibody) units of cells were separated by SDS-PAGE followed by Western blotting and immunodecoration. Signal detection was performed using an enhanced chemiluminescent (ECL) detection system (GE Healthcare) or Immunostar Zeta (Wako). The anti-Atg13 antibody was a gift from Dr. Ohsumi (Tokyo Institute of Technology, Japan). Anti-GFP (NACALAI TESQUE), anti-Pgk1 (Invitrogen), PAP (Sigma Aldrich), and anti-myc (Cell Signaling Technology) antibodies were used to detect GFP, Pgk1, TAP, and myc, respectively.

### Real-time quantitative RT–PCR

RNA samples were prepared by the hot-phenol assay using phenol∶chloroform (5∶1; pH 4.7) (Sigma Aldrich) and the Qiagen RNeasy Mini Kit (Qiagen). The reverse transcriptase reaction was performed using the ReverTra Ace qPCR RT Kit (TOYOBO) or the ReverTra Ace qPCR RT Master Mix with gDNA Remover (TOYOBO). Real-time PCR was performed using SYBR Green Realtime PCR Master Mix Plus (TOYOBO) in duplicate. The mRNA levels of *RPS26A*, *RPL9A*, *MEP2*, *GAP1*, and *NOG1* were quantified using corresponding primer sets purchased from OPERON. *TUB1* and *ACT1* were used as controls for quantification.

### Cell synchrony and fluorescence-activated cell sorter (FACS) analysis

Cell synchronization in G1 was obtained by treating exponentially growing *MAT* **a** cells with 4 ng/mL α-factor mating pheromone for ∼2 h. G1-arrested cells were washed with distilled water twice and conditioned medium once, and released into fresh SCD medium. After 0.75 h, synchronized cells were washed with distilled water three times, released into fresh SD-N and SCD medium with or without 200 ng/mL rapamycin (Sigma Aldrich) and 6.7 ng/mL α-factor, and collected at the indicated times. For cell synchronization at metaphase, G1-synchronized cells were washed with distilled water three times, released into fresh SD-N medium containing 5 µg/mL nocodazole (Sigma Aldrich) after 0.75 h from α-factor release. After 3 h, synchronous cultures arrested at metaphase by nocodazole were collected and re-released into SD-N medium with or without 1 mM PMSF (Wako), and collected at the indicated times.

For FACS analysis, cells were fixed with 70% ethanol at 4°C overnight. Then the cells were washed and suspended in 50 mM sodium citrate, treated with 250 µg/mL RNase A at 50°C for 1 h, and then treated with 1 mg/mL proteinase K at 50°C for 1 h. The resuspended cells were stained with 16 µg/mL propidium iodide at room temperature for 30 min. The DNA content of cells was measured on a Beckman-Coulter flow cytometer.

### Examination of nuclear DNA

Nuclear DNA was visualized by the addition of 1 µg/mL Hoechst 33342 or 50 ng/mL DAPI after cells were fixed with 3% formaldehyde at 4°C overnight. For staining by DAPI, cells fixed with formaldehyde were washed with PBS twice, and suspended in PBS with 0.1% Triton-X100. After 1 h, cells were washed with PBS 5 times, and suspended in PBS. Cells were observed using an inverted microscope (Delta Vision or Leica DMI 4000B). Images were captured using image acquisition and analysis software.

### Quantification of aneuploidy formation

Quantification of aneuploidy formation was performed using the system developed by Chan and Botstein [39]. This assay is based on a genetic system that detects yeast cells with extra copies of a genetically marked chromosome. To monitor the frequency of aneuploidy, appropriately diluted cell cultures were plated onto yeast extract peptone dextrose (YEPD) plates and selective medium lacking both leucine and uracil. The number of colonies was scored after incubation for 4 days at 30°C.

### Statistic analysis of diameter ratios

Data sets of diameter ratios were analyzed as described previously [63]. First, the data were subjected to the Kruskal-Wallis rank sum test, with *P*<0.05 considered significant. Then, the significance of differences between WT and Δ*atg2* cells was determined using Sokal and Rohlf's test of nonparametric multiple comparisons by STP [64]. Equal-sized subsets were obtained from the larger data subsets using a random selection algorithm.

## Supporting Information

Figure S1 Validation of *TUB1* as a control for quantification of RT-qPCR under starvation conditions. *ATG2* (SAY122) and Δ*atg2* (AMY250) cells were grown as described in Figure 2A. Total RNA was extracted and analyzed for the expression of *ACT1* (left panel) and *TUB1* (right panel) by RT-qPCR. Each sample was calibrated by *TUB1* and *ACT1*, respectively.
(PDF)

Figure S2 Atg1 is required for cell cycle progression during starvation. *ATG1* (SAY122) and Δ*atg1* (AMY240) cells were arrested at G1 by α-factor and released into SCD medium. Synchronous cultures were collected at 0.75 h and re-released into SD-N medium or SCD medium. To monitor cell cycle progression to G1 under the nutrient-rich condition, SCD medium was supplemented with 6.7 ng/mL α-factor for re-arrest at G1. After 25 h, the cell cultures were re-released into SCD medium. DNA content at each time point was measured by FACS analysis.
(PDF)

Figure S3 Recovery of TORC1 activity may be dispensable for cell cycle progression during starvation conditions. (A) *KOG1* (YYK409) and *kog1-105* (YYK410) cells were arrested at G1 by α-factor and released into SCD medium at 25°C. Synchronous cultures were collected after 0.75 h and re-released into SD-N medium or SCD medium at 25°C. To monitor cell cycle progression to G1 under the nutrient-rich condition, SCD medium was supplemented with 6.7 ng/mL α-factor for re-arrest at G1. DNA content was measured by FACS analysis. (B) *KOG1* (YYK409) and *kog1-105* (YYK410) cells were grown as described in (A). Total RNA was extracted and analyzed for the expression of *RPS26A* (upper panel) and *MEP2* (lower panel) by RT-qPCR. Each sample was calibrated by *TUB1*. Values represent the mean ± SEM (n = 3; *p<0.001, Student's t test). (C) *KOG1* (YYK409) and *kog1-105* (YYK410) cells were arrested at G1 by α-factor and released into SCD medium at 25°C. Synchronous cultures were collected after 0.75 h and re-released into SD-N medium or SD-N medium containing rapamycin. DNA content was measured by FACS analysis. Rapamycin was used at a final concentration of 200 ng/mL.
(PDF)

Figure S4 Autophagy is important for efficient recovery from the Swe1-dependent checkpoint under starvation conditions. Δ*swe1* (AMY260) and Δ*atg2* Δ*swe1* (AMY261) cells were arrested at G1 with α-factor and released into SCD medium. Synchronous cultures were collected after 0.75 h and re-released into SD-N medium or SCD medium. To monitor cell cycle progression to G1 under the nutrient-rich condition, SCD medium was supplemented with 6.7 ng/mL α-factor for re-arrest at G1. DNA content was measured by FACS analysis.
(PDF)

Figure S5 Full scan of the immunoblots shown in Figure 1, Figure 2, and Figure 6.
(PDF)

Table S1 Strains used in this study.
(PDF)
