# Supplementary figures and images for: The Role of Autophagy in Genome Stability through Suppression of Abnormal Mitosis under Starvation
Source: PLoS Genet. 2013 Jan 31;9(1):e1003245. doi: 10.1371/journal.pgen.1003245 (PMC3561091; doi:10.1371/journal.pgen.1003245)

# Figure S1

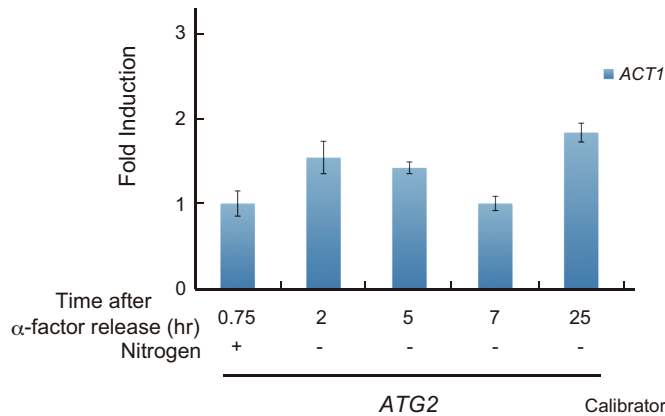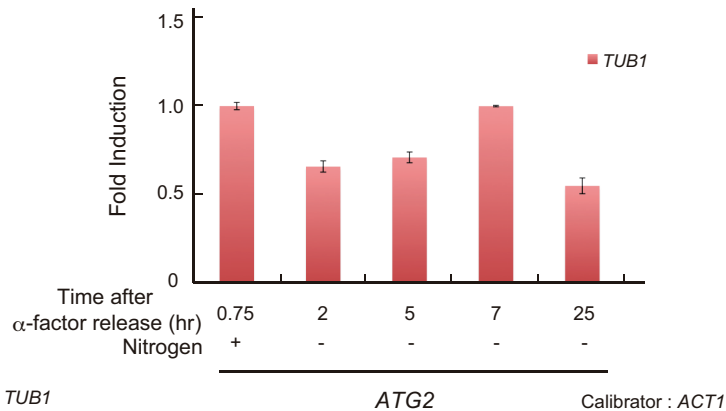

Supplement: Figure S1 — Validation of TUB1 as a control for quantification of RT-qPCR under starvation conditions. ATG2 (SAY122) and Δatg2 (AMY250) cells were grown as described in Figure 2A. Total RNA was extracted and analyzed for the expression of ACT1 (left panel) and TUB1 (right panel) by RT-qPCR. Each sample was calibrated by TUB1 and ACT1, respectively. (PDF) [file pgen.1003245.s001.pdf]

Figure S2

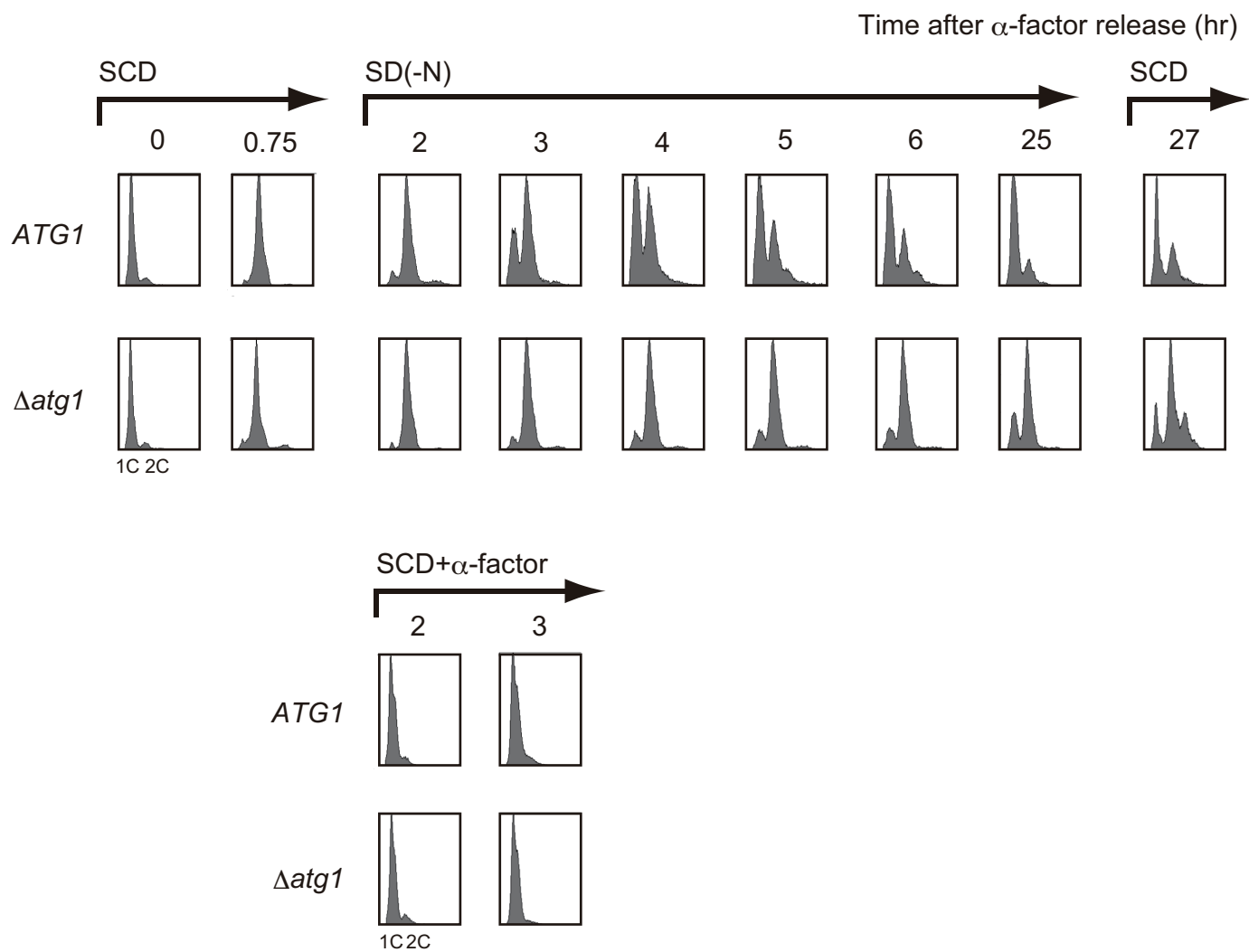

Supplement: Figure S2 — Atg1 is required for cell cycle progression during starvation. ATG1 (SAY122) and Δatg1 (AMY240) cells were arrested at G1 by α-factor and released into SCD medium. Synchronous cultures were collected at 0.75 h and re-released into SD-N medium or SCD medium. To monitor cell cycle progression to G1 under the nutrient-rich condition, SCD medium was supplemented with 6.7 ng/mL α-factor for re-arrest at G1. After 25 h, the cell cultures were re-released into SCD medium. DNA content at each time point was measured by FACS analysis. (PDF) [file pgen.1003245.s002.pdf]

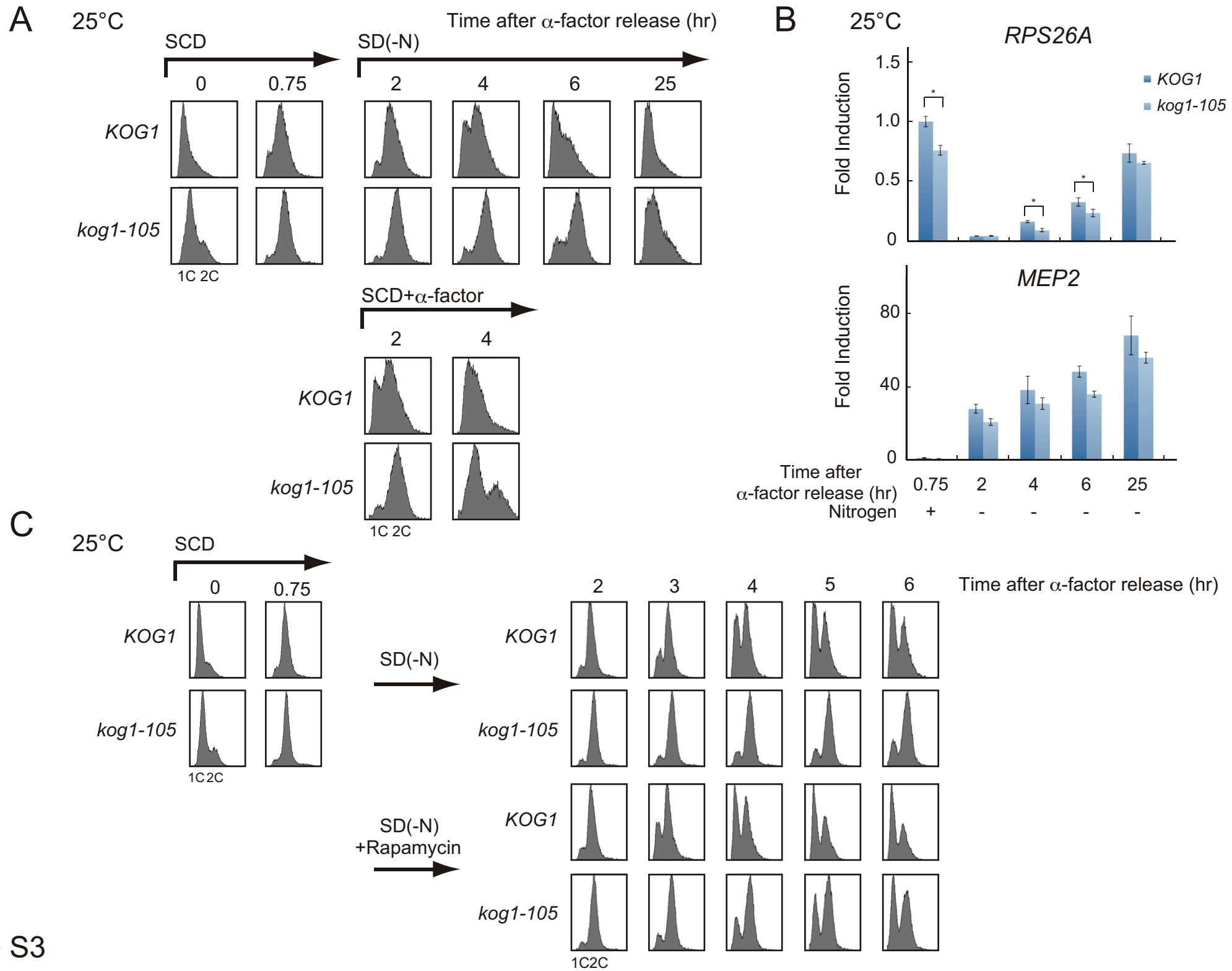

Figure S3

Supplement: Figure S3 — Recovery of TORC1 activity may be dispensable for cell cycle progression during starvation conditions. (A) KOG1 (YYK409) and kog1-105 (YYK410) cells were arrested at G1 by α-factor and released into SCD medium at 25°C. Synchronous cultures were collected after 0.75 h and re-released into SD-N medium or SCD medium at 25°C. To monitor cell cycle progression to G1 under the nutrient-rich condition, SCD medium was supplemented with 6.7 ng/mL α-factor for re-arrest at G1. DNA content was measured by FACS analysis. (B) KOG1 (YYK409) and kog1-105 (YYK410) cells were grown as described in (A). Total RNA was extracted and analyzed for the expression of RPS26A (upper panel) and MEP2 (lower panel) by RT-qPCR. Each sample was calibrated by TUB1. Values represent the mean ± SEM (n = 3; *p<0.001, Student's t test). (C) KOG1 (YYK409) and kog1-105 (YYK410) cells were arrested at G1 by α-factor and released into SCD medium at 25°C. Synchronous cultures were collected after 0.75 h and re-released into SD-N medium or SD-N medium containing rapamycin. DNA content was measured by FACS analysis. Rapamycin was used at a final concentration of 200 ng/mL. (PDF) [file pgen.1003245.s003.pdf]

Figure S4

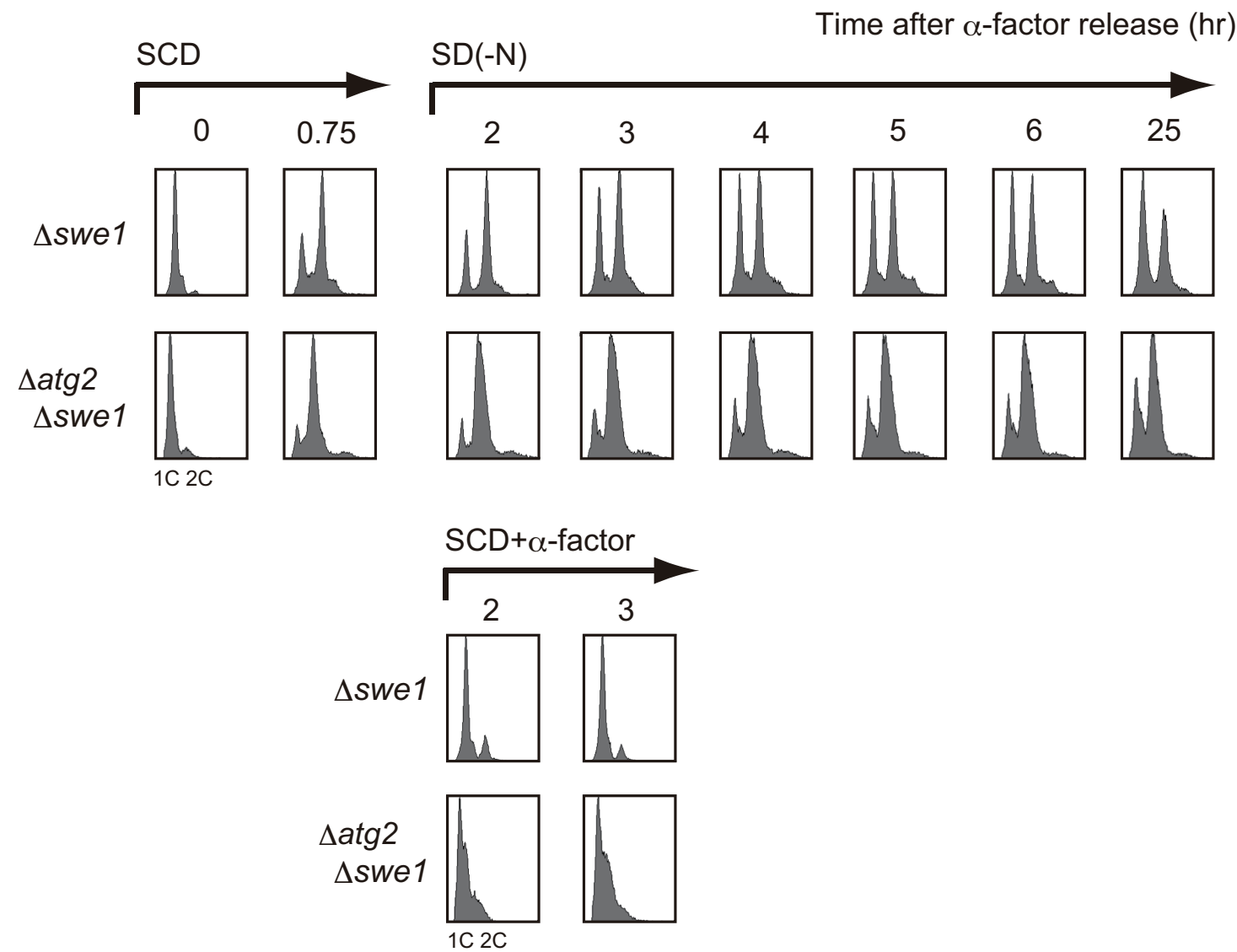

Supplement: Figure S4 — Autophagy is important for efficient recovery from the Swe1-dependent checkpoint under starvation conditions. Δswe1 (AMY260) and Δatg2 Δswe1 (AMY261) cells were arrested at G1 with α-factor and released into SCD medium. Synchronous cultures were collected after 0.75 h and re-released into SD-N medium or SCD medium. To monitor cell cycle progression to G1 under the nutrient-rich condition, SCD medium was supplemented with 6.7 ng/mL α-factor for re-arrest at G1. DNA content was measured by FACS analysis. (PDF) [file pgen.1003245.s004.pdf]

# Figure S5

Figure 1A

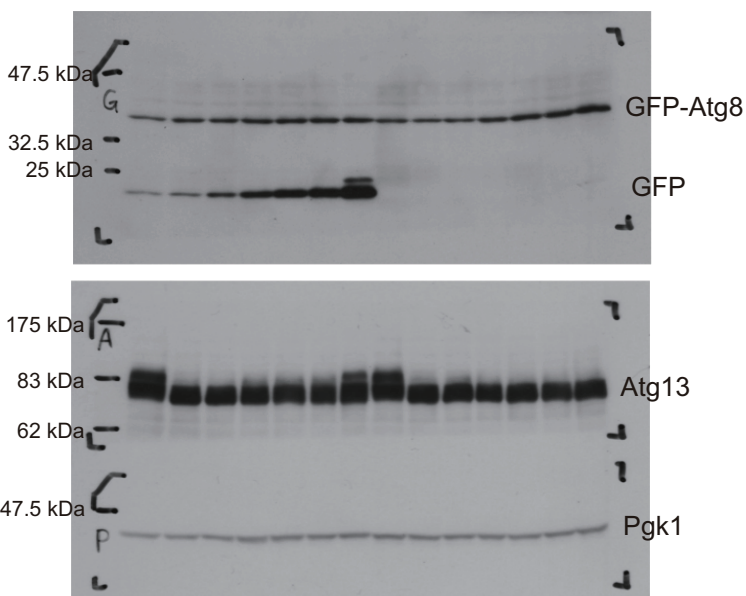

Figure 1C

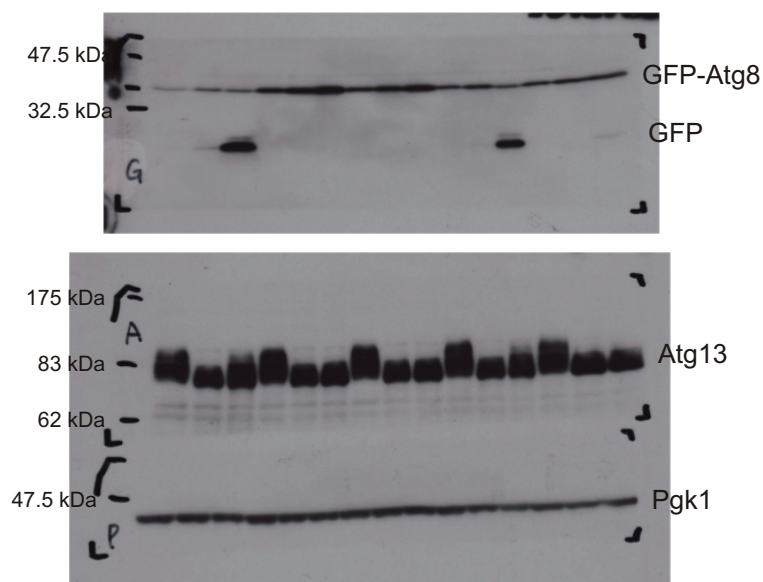

Figure 2B

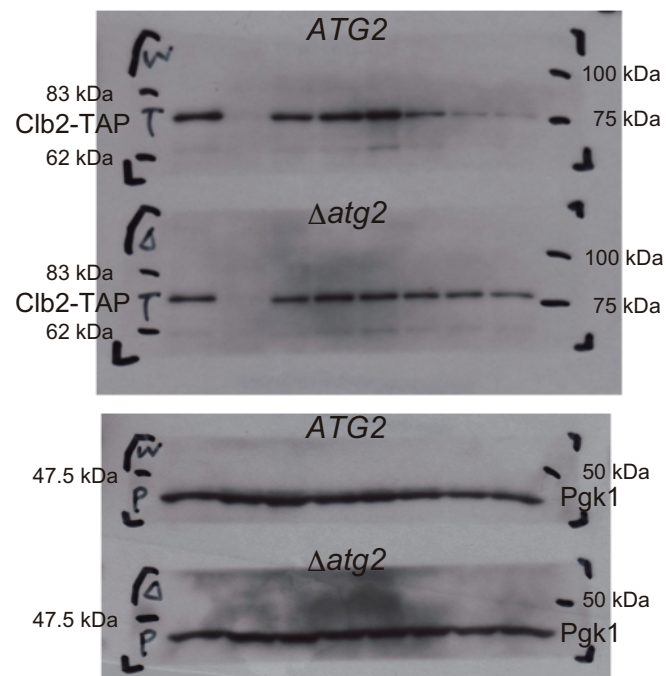

Figure 6C

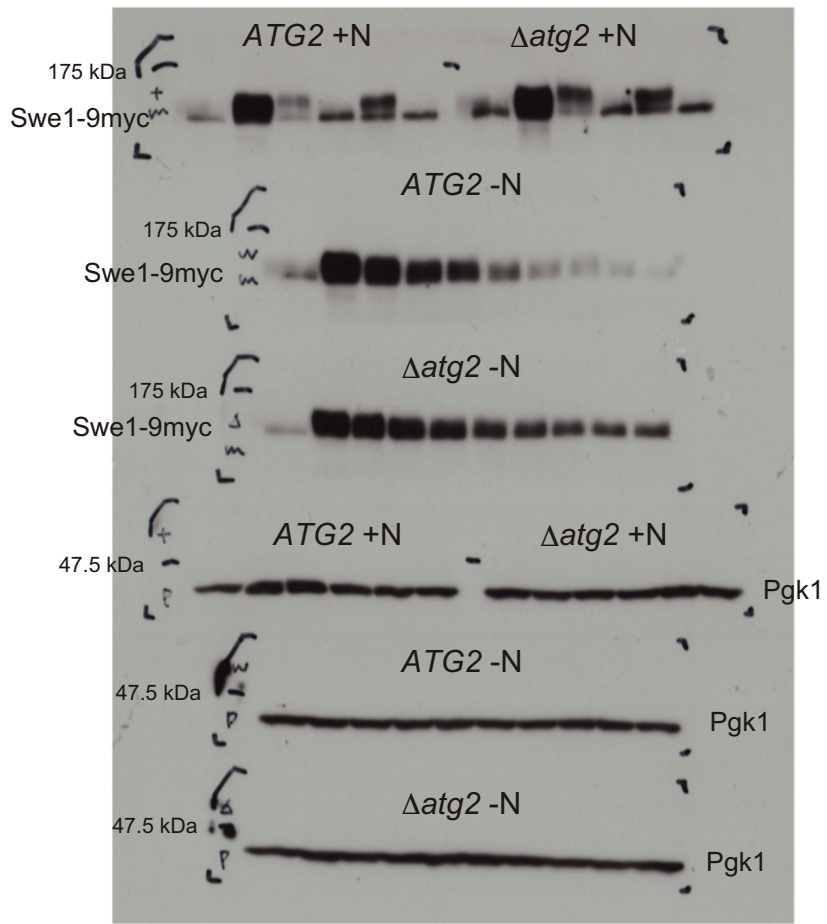

Supplement: Figure S5 — Full scan of the immunoblots shown in Figure 1, Figure 2, and Figure 6. (PDF) [file pgen.1003245.s005.pdf]
