# Supplementary material for: The Role of Autophagy in Genome Stability through Suppression of Abnormal Mitosis under Starvation
Source: PLoS Genet. 2013 Jan 31;9(1):e1003245. doi: 10.1371/journal.pgen.1003245 (PMC3561091; doi:10.1371/journal.pgen.1003245)
Supplement: Table S1 — Strains used in this study. (PDF) [file pgen.1003245.s006.pdf]

Table S1. Yeast strains used in this study

Strain	Genotype	Source
W303-1B	*MAT***a** *ade2 ura3 trp1 his3 leu2*	Laboratory stock
W303-1A	*MATα ade2 ura3 trp1 his3 leu2*	Laboratory stock
SAY122	*MAT***a** *ura3 trp1 his3 leu2 TUB1-GFP*::*LEU2*	Laboratory stock
DBY4962	*MAT***a** *ade2 his3*-Δ*200 ura3-52 leu2*-Δ*101*::*URA3*::*leu2*-Δ*102 lys2*-Δ*101*::*HIS3*::*lys2*-Δ*102*	[39]
AMY182-10A	*MATα ade2 ura3 trp1 his3 leu2 atg2*::*kanMX* pRS314-*GFP-ATG8* YEp352-*ATG13*	Laboratory stock
AMY182-10C	*MAT***a** *ade2 ura3 trp1 his3 leu2* pRS314-*GFP-ATG8* YEp352-*ATG13*	Laboratory stock
AMY236	*MAT***a** *ade2 ura3 trp1 his3 leu2 atg1*::*kanMX* pRS314-*GFP-ATG8* YEp352-*ATG13*	This study
AMY237	*MAT***a** *ade2 ura3 trp1 his3 leu2 atg7*::*kanMX* pRS314-*GFP-ATG8* YEp352-*ATG13*	This study
AMY238	*MAT***a** *ade2 ura3 trp1 his3 leu2 atg11*::*kanMX* pRS314-*GFP-ATG8* YEp352-*ATG13*	This study
AMY239	*MAT***a** *ade2 ura3 trp1 his3 leu2 pep4*::*kanMX* pRS314-*GFP-ATG8* YEp352-*ATG13*	This study
AMY240	*MAT***a** *ura3 trp1 his3 leu2 TUB1-GFP*::*LEU2 atg1*::*kanMX*	This study
AMY250	*MAT***a** *ura3 trp1 his3 leu2 TUB1-GFP*::*LEU2 atg2*::*kanMX*	This study
AMY251	*MAT***a** *ade2 ura3 trp1 his3 leu2 CLB2-TAP*::*His3MX*	This study
AMY253	*MAT***a** *ade2 ura3 trp1 his3 leu2 atg2*::*kanMX CLB2-TAP*::*His3MX*	This study
AMY255	*MAT***a** *ura3 trp1 his3 leu2 TUB1-GFP*::*LEU2 atg2*::*HygMX*	This study
AMY260	*MAT***a** *ura3 trp1 his3 leu2 TUB1-GFP*::*LEU2 swe1*::*kanMX*	This study
AMY261	*MAT***a** *ura3 trp1 his3 leu2 TUB1-GFP*::*LEU2 atg2*::*HygMX swe1*::*kanMX*	This study
AMY262	*MAT***a** *ade2 his3*-Δ*200 ura3-52 leu2*-Δ*101*::*URA3*::*leu2*-Δ*102 lys2*-Δ*101*::*HIS3*::*lys2*-Δ*102 atg2*::*kanMX*	This study
AMY296	*MAT***a** *ade2 ura3 trp1 his3 leu2 atg2*::*KanMX*	This study
AMY330	*MAT***a** *ade2 ura3 trp1 his3 leu2 swe1*::*HIS3*::*SWE1-9myc*::*URA3 atg2*::*KanMX*	This study
YYK409	*MAT***a** *ade2 ura3 trp1 his3 leu2 kog1*::*LEU2* pRS313-*KOG1*	[20]
YYK410	*MAT***a** *ade2 ura3 trp1 his3 leu2 kog1*::*LEU2* pRS313-*kog1-105*	[20]
YYK536	*MAT***a** *ade2 ura3 trp1 his3 leu2 swe1*::*HIS3*::*SWE1-9myc*::*URA3*	[66]
